# First Report of *Chlamydia* Seroprevalence and Risk Factors in Domestic Black-Boned Sheep and Goats in China

**DOI:** 10.3389/fvets.2020.00363

**Published:** 2020-07-17

**Authors:** Li-Xiu Sun, Qin-Li Liang, Xiao-Hui Hu, Zhao Li, Jian-Fa Yang, Feng-Cai Zou, Xing-Quan Zhu

**Affiliations:** ^1^State Key Laboratory of Veterinary Etiological Biology, Lanzhou Veterinary Research Institute, Chinese Academy of Agricultural Sciences, Lanzhou, China; ^2^Key Laboratory of Veterinary Public Health of Yunnan Province, College of Veterinary Medicine, Yunnan Agricultural University, Kunming, China

**Keywords:** *Chlamydia*, domestic black-boned sheep and goats, indirect hemagglutination assay, seroprevalence, China

## Abstract

The Gram-negative bacteria of the genus *Chlamydia* cause a wide range of diseases in humans and animals. The seroprevalence of *Chlamydia* in domestic black-boned sheep and goats in China is unknown. In this survey, a total of 481 serum samples were collected randomly from domestic black-boned sheep and goats from three counties in Yunnan province, southwest China, from July to August 2017. The sera were examined by an indirect hemagglutination assay (IHA). Antibodies to *Chlamydia* were detected in 100/481 [20.79%, 95% confidence interval (CI), 17.16–24.42] serum samples (IHA titer ≥1:64). The *Chlamydia* seroprevalence ranged from 12.21% (95% CI, 7.81–16.61) to 30.89% (95% CI, 22.72–39.06) across different regions in Yunnan province, and the differences were statistically significant (*P* < 0.01). The seroprevalence in male domestic black-boned sheep and goats (28.64%; 95% CI, 22.36–34.92) was significantly higher than that in the females (15.25%; 95% CI, 11.05–19.45) (*P* < 0.01). However, there was no statistically significant difference in *Chlamydia* seroprevalence in domestic black-boned sheep and goats between ages and species (*P* > 0.05). To our knowledge, this is the first report of *Chlamydia* seroprevalence in domestic black-boned sheep and goats in Yunnan Province, southwest China. These data provide baseline information for future implementation of measures to control *Chlamydia* infection in these animals.

## Introduction

*Chlamydia*, an obligate intracellular Gram-negative pathogen, is responsible for a broad spectrum of diseases in animals and humans (1, 2). *Chlamydia* grows and reproduces in the respiratory, urogenital, and gastrointestinal tracts (2). Two species of the genus *Chlamydia*, namely *Chlamydia abortus* and *Chlamydia pecorum*, can cause serious infections in sheep and goats (1). *Chlamydia* is a leading cause of abortion in sheep and goats, which caused significant economic losses to livestock industry (3–6). Additionally, as a zoonotic pathogen, humans can be infected via exposure to *Chlamydia* infected animals (7).

*Chlamydia* infection is prevalent in sheep and goats all over the world, especially in sheep-rearing areas, such as in Northern Europe and North America (8, 9). In China, *Chlamydia* infection in sheep has been reported in many provinces, such as Qinghai, Shandong, and Hubei (10). However, data about *Chlamydia* infection in domestic black-boned sheep and goats have been limited. Domestic black-boned sheep and goats have dark tissue compared to ordinary sheep and goats, which has been attributed to the presence of excessive melanin in domestic black-boned sheep and goats (11).

Domestic black-boned sheep and goats are indigenous animals to the Lanping County of Yunnan Province, China (11–13). Because of their unique characteristics of these breeds, black-boned sheep and goats have a strong adaptability, and they have been introduced into other provinces of China, such as Shandong, Henan, and Hebei (14). Therefore, in this study, we examined the seroprevalence and risk factors of *Chlamydia* infection in domestic black-boned sheep and goats in Yunnan Province, southwest China. Our results provide baseline data for future control strategies of *Chlamydia* infection in domestic black-boned sheep and goats in China.

## Materials and Methods

### Ethical Statement

This study was approved by the Animal Administration and Ethics Committee of Lanzhou Veterinary Research Institute, Chinese Academy of Agricultural Sciences (approval no.: LVRIAEC-2017-06). Domestic black-boned sheep and goats, from which the blood samples were collected, were handled humanely in accordance with the requirements of the Animal Ethics Procedures and Guidelines of the People's Republic of China.

### The Study Sites

**Figure 1:**
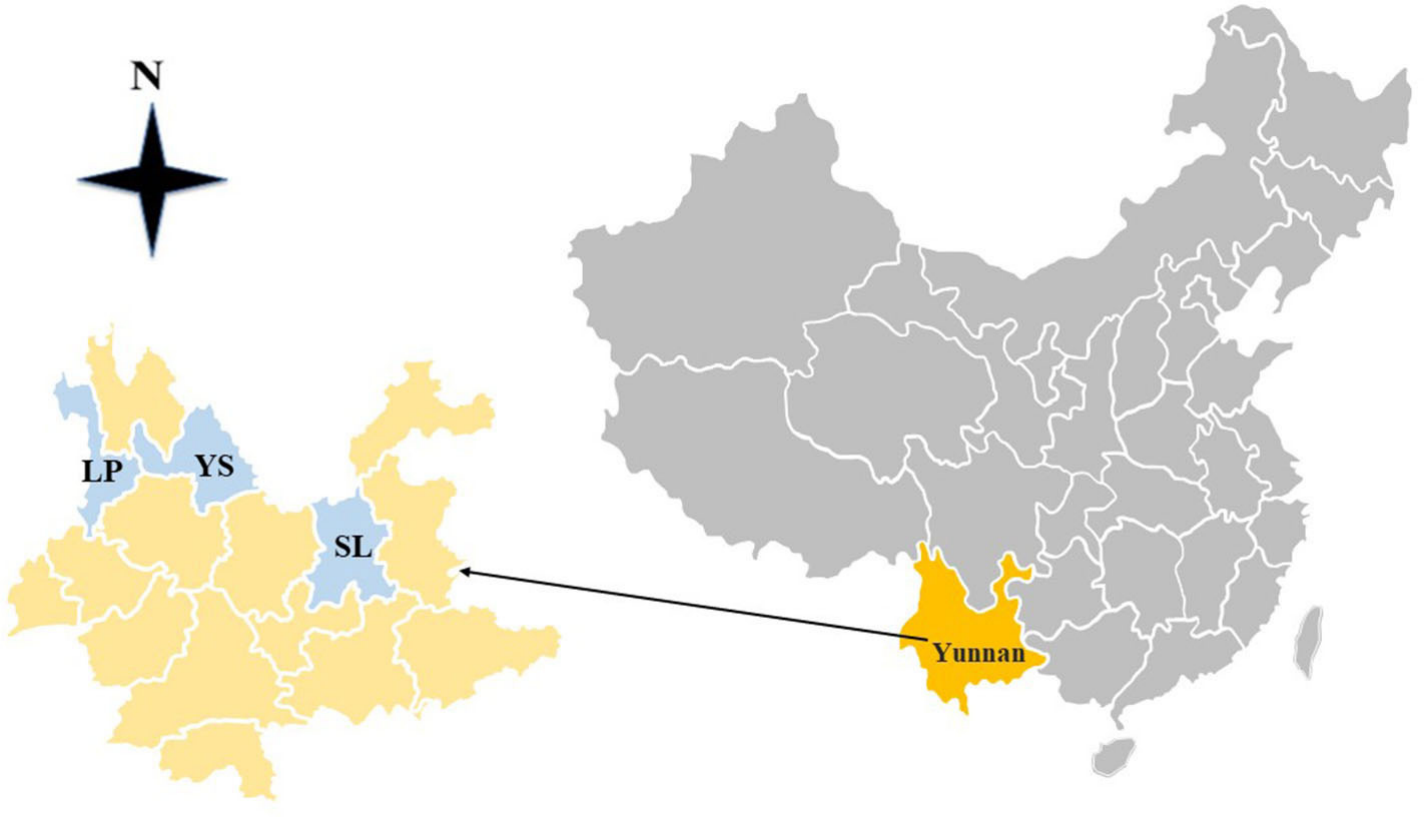
The map of China showing the geographical regions in Yunnan province, where domestic black-boned sheep and goats were sampled. LP, Lanping County; SL, Shilin County; YS, Yongsheng County.

The survey was conducted in Shilin County, Lanping County, and Yongsheng County in Yunnan Province, southwest China (Figure 1). Yunnan Province is the major producing region of domestic black-boned sheep and goats in China. In the present study, the sampling sites are all large-scale farms, which implement a free-range breeding mode for 5–8 h in daytime. The annual temperature difference in Yunnan Province is small, but the daily temperature difference is large.

### Serum Samples

Between July and August 2017, a total of 481 blood samples were collected randomly from domestic black-boned sheep and goats from four intensive farms (*n* = 6,100), two of which were from Lanping county (*n* = 213), followed by Yongsheng county (*n* = 145) and Shilin county (*n* = 123), Yunnan province, southwest China. A standardized questionnaire was used to collect information about the region, gender, age, and species of each animal. Blood samples were transported to the laboratory, kept at room temperature for 2 h, and centrifuged at 3,000 g for 10 min, and the supernatants, which represent the serum samples, were collected and stored at −20°C until further analysis.

### Serological Examination

A commercially available indirect hemagglutination assay (IHA) kit (Lanzhou Veterinary Research Institute, Chinese Academy of Agricultural Sciences) was used to determine the level of *Chlamydia* antibodies in the serum of domestic black-boned sheep and goats. As a mature technology for detecting *Chlamydia* antibodies, the sensitivity and specificity of the IHA kit used in this study have been verified by the Ministry of Agriculture of China (NY/T 562-2002), which were 100% and 95%, respectively (15). The serological analysis was carried out according to the manufacturer's recommendations as previously described (16–19). Briefly, serum samples were added to 96-well V-bottomed polystyrene plates, which were diluted fourfold serially from 1:4 to 1:1,024. The detection antigen was added to each well, and the plate was then shaken slightly for 2 min followed by incubation at 37°C for 2 h. The samples were considered positive for *Chlamydia* antibodies when the agglutinated erythrocytes were formed in wells at dilutions of 1:64 or higher. Samples that had agglutination results between 1:4 and 1:64 were considered “suspect” and were retested.

### Statistical Analysis

Differences in the seroprevalence of *Chlamydia* among domestic black-boned sheep and goats of different regions, genders, ages, and species were analyzed by a χ^2^ test using the SPSS software (release 23.0 standard version; SPSS Inc., Chicago, IL, USA). *P* < 0.05 was considered statistically significant. Odds ratios (ORs) with 95% confidence intervals (CIs) were also determined.

## Results

In the present study, 100 of the examined 481 serum samples of domestic black-boned sheep and goats (20.79%; 95% CI, 17.16–24.42) were seropositive for *Chlamydia* by IHA test at the cutoff titer of 1:64. The 100 positive samples included 26 samples (of 213) from Lanping Country (12.21%; 95% CI, 7.81–16.61), 36 (of 145) from Yongsheng Country (24.83%; 95% CI, 17.80–31.86), and 38 (of 123) samples from Shilin Country (30.89%; 95% CI, 22.72–39.06). The differences in *Chlamydia* seroprevalence between these regions were statistically significant (χ^2^ = 18.59, *df* = 2, *P* < 0.01; Table 1). As shown in Table 1, the investigation revealed that the seroprevalence in female and male animals was 15.25% (43/282; 95% CI, 11.05–19.45) and 28.64% (57/199; 95% CI, 22.36–34.92), respectively. The difference in *Chlamydia* seroprevalence was statistically significant between genders (χ^2^ = 12.71, *df* = 1, *P* < 0.01) of domestic black-boned sheep and goats. Seropositive black-boned sheep and goats were found in all four age groups and varied from 16.41% (21/128; 95% CI, 10.00–22.83) to 25.40% (48/189; 95% CI, 19.19–31.61). In terms of species, the seroprevalence was 22.76% (71/312; 95% CI, 18.11–27.41) in black-boned sheep and 17.16% (29/169; 95% CI, 11.48–22.84) in black-boned goats. There was no statistically significant difference in *Chlamydia* seroprevalence observed between age groups (χ^2^ = 4.63, *df* = 3, *P* > 0.05) and species *(*χ^2^ = 2.09, *df* = 1, *P* > 0.05) in domestic black-boned sheep and goats (Table 1). The antibody titers were diverse in domestic black-boned sheep and goats of different regions, genders, ages, and species, with the most frequent titers being 1:64 (87.00%), followed by 1:256 (10.00%) and 1:1,024 (3.00%; Table 1).

**Table 1:** Seroprevalence and risk factors for *Chlamydia* in domestic black-boned sheep and goats in Yunnan Province, southwest China, determined by indirect hemagglutination (IHA) test.

**Variables**	**Categories**	**Antibody titers**			**No. tested**	**No. positive**	**Prevalence (%) (95% CI)**	**OR (95% CI)**	***P*-value**
		**1:64**	**1:256**	**1:1.024**					
Region	Lanping	22	1	3	213	26	12.21 (7.81–16.61)	Reference	<0.01
	Yongsheng	33	3	0	145	36	24.83 (17.80–31.86)	2.38 (1.36–4.15)	
	Shilin	32	6	0	123	38	30.89 (22.72–39.06)	3.22 (1.84–5.63)	
Gender	Female	40	1	2	282	43	15.25 (11.05–19.45)	Reference	<0.01
	Male	47	9	1	199	57	28.64 (22.36–34.92)	2.23 (1.43–3.49)	
Age (years)^a^	<0 to ≤ 1	42	6	0	189	48	25.40 (19.19–31.61)	1.73 (0.98–3.07)	0.2013
	1 < to ≤ 2	17	1	0	103	18	17.48 (10.15–24.81)	1.08 (0.54–2.15)	
	2 < to ≤ 3	18	1	2	128	21	16.41 (10.00–22.83)	Reference	
	>3	10	2	1	61	13	21.31 (11.03–31.59)	1.38 (0.64–2.98)	
Species	BBG^b^	28	0	1	169	29	17.16 (11.48–22.84)	Reference	0.1487
	BBS^c^	59	10	2	312	71	22.76 (18.11–27.41)	1.42 (0.88–2.30)	
	Total	87	10	3	481	100	20.79 (17.16–24.42)		

a *Year*.

b *Domestic black-boned goat*.

c *Domestic black-boned sheep*.

*OR, odds ratio; CI, confidence interval*.

## Discussion

In this study, the seroprevalence of *Chlamydia* in domestic black-boned sheep and goats in Yunnan province was 20.79%, which was higher than the 8.45% reported in goats in Hunan Province, China (20), but was lower than that reported in sheep in Xinjiang Province (40.13%) in China (10). *Chlamydia* seroprevalence has been reported in sheep and goats worldwide. For example, 10.60% seroprevalence has been reported in sheep in India (21), and 33% seroprevalence has been reported in Spain (22). The different seroprevalences in different counties in our study is probably attributed to the differences in sanitation, husbandry practices, and animal welfare. In addition, other reasons for the variations of prevalence may include different ecological and geographical factors including temperature, rainfall, altitude, or level of vegetation. Furthermore, differences in the serological methods and cutoff titers used may be other factors that influence the seroprevalence of *Chlamydia* in different regions.

The overall *Chlamydia* seroprevalence in domestic black-boned sheep and goats in Shilin County was 30.89%, which was higher than the seroprevalence in Yongsheng County (24.83%) and in Lanping County (12.21%). There was significant difference in *Chlamydia* seroprevalence in domestic black-boned sheep and goats of different regions (*P* < 0.01). This result is consistent with a previous study that reported an 18.65% *Chlamydia* seroprevalence in Tibetan sheep in Gansu province (15). *Chlamydia* is significantly resistant under dry, cold (5–10°C), and dark conditions (23). Yunnan Province has a generally mild climate as diverse as its terrain. Shilin Country has an average annual temperature of 15°C and a mean annual rainfall of 1,010 mm. The warm temperature and appropriate precipitation in Shilin Country are favorable for the survival of *Chlamydia*. Therefore, the differences in *Chlamydia* seroprevalence in domestic black-boned sheep and goats across different regions are probably attributed to the variable climatic conditions in Yunnan Province.

Statistically, the *Chlamydia* seroprevalence in male (28.64%) domestic black-boned sheep and goats was significantly higher than in the females (15.25%). Statistical analysis showed a significant difference between genders (*P* < 0.01). Gender-related differences in *Chlamydia* seroprevalence were related to variations in immune response or antibody persistence between males and females (24). The result was different from a previous study, which reported no effect of the gender on the prevalence of *Chlamydia* infection in sheep (17).

The seroprevalence of *Chlamydia* varied across the different age groups of domestic black-boned sheep and goats. The highest seroprevalence was 25.40% in black-boned sheep and goats of the 0 < years ≤ 1 age group, and the lowest prevalence was 16.41% in the 2 < years ≤ 3 age group. But the differences were not statistically significant among different age groups (*P* > 0.05), which disagree with the study of Qin et al. (15), which reported positive association of *Chlamydia* seroprevalence with the ages of Tibetan sheep in Gansu Province. The higher seroprevalence in domestic black-boned sheep and goats of the 0 < years ≤ 1 age group may be due to the low levels of antibodies, which makes them more susceptible to infection. The different prevalence in different age groups indicates the possibility of horizontal transmission in investigated black-boned sheep and goat herds (25).

The seroprevalence of *Chlamydia* in domestic black-boned sheep (22.76%) was slightly higher than that in domestic black-boned goats (17.16%), which may be related to the different susceptibility of goats and sheep to *Chlamydia*. Statistical analysis suggested that species may not be a crucial factor for *Chlamydia* infection in black-boned sheep and goats. The difference in *Chlamydia* seroprevalence in domestic black-boned sheep and goats may be caused by the sample bias, where more domestic black-boned sheep samples were examined than black-boned goats.

There are some limitations to the present investigation. The serum samples of black-boned sheep and goats examined in this study were collected from July to August 2017, a relatively short sampling time; thus, the reported *Chlamydia* seroprevalence may not fully reflect the true situation of long-term infection of *Chlamydia* in domestic black-boned sheep and goats. Given that domestic black-boned sheep and goats have been introduced into other provinces of China (14), further research should investigate *Chlamydia* seroprevalence in domestic black-boned sheep and goats in these provinces, which will provide global baseline data for the prevention of *Chlamydia* infection in black-boned sheep and goats in China.

## Conclusion

The present study revealed that *Chlamydia* seroprevalence (20.79%) is relatively high in domestic black-boned sheep and goats in Yunnan Province, southwest China. This study also demonstrated that region and gender are the main risk factors for *Chlamydia* seroprevalence between domestic black-boned sheep and goats. To our knowledge, the present survey is the first to document the seroprevalence of *Chlamydia* infection in domestic black-boned sheep and goats in China, which provided baseline data for future prevention and control of *Chlamydia* in domestic black-boned sheep and goats.

## Data Availability Statement

All datasets generated for this study are included in the article.

## Ethics Statement

The animal study was reviewed and approved by The Animal Ethics and Administration Committee of Lanzhou Veterinary Research Institute, Chinese Academy of Agricultural Sciences.

## Author Contributions

X-QZ and F-CZ conceived and designed the experiments. L-XS performed the experiments, analyzed the data, and wrote the paper. ZL, J-FY, and F-CZ participated in the collection of serum samples. Q-LL and X-HH participated in the implementation of the study. X-QZ and F-CZ critically revised the manuscript. All authors have read and approved the final version of the manuscript. All authors contributed to the preparation of the manuscript.

## Conflict of Interest

The authors declare that the research was conducted in the absence of any commercial or financial relationships that could be construed as a potential conflict of interest.
